# Development of novel monoclonal antibodies against nsp12 of SARS-CoV-2

**DOI:** 10.1186/s12985-022-01948-2

**Published:** 2022-12-10

**Authors:** Mitsuhiro Machitani, Junko Takei, Mika K. Kaneko, Saori Ueki, Hirofumi Ohashi, Koichi Watashi, Yukinari Kato, Kenkichi Masutomi

**Affiliations:** 1 Division of Cancer Stem Cell, National Cancer Center Research Institute, 5-1-1 Tsukiji, Chuo-Ku, Tokyo, 104-0045 Japan; 2 Department of Antibody Drug Development, Tohoku University Graduate School of Medicine, 2-1 Seiryo-Machi, Aoba-Ku, Sendai, Miyagi 980-8575 Japan; 3 Research Center for Drug and Vaccine Development, National Institute of Infectious Diseases, 1-23-1 Toyama, Shinjuku, Tokyo, 162-8640 Japan; 4 Department of Virology II, National Institute of Infectious Diseases, 1-23-1 Toyama, Shinjuku, Tokyo, 162-8640 Japan; 5 Department of Molecular Pharmacology, Tohoku University Graduate School of Medicine, 2-1 Seiryo-Machi, Aoba-Ku, Sendai, Miyagi 980-8575 Japan

**Keywords:** SARS-CoV-2, RNA-dependent RNA polymerase, nsp12, Monoclonal antibody

## Abstract

A novel coronavirus, severe acute respiratory syndrome coronavirus 2 (SARS-CoV-2), has caused a global pandemic of coronavirus disease 19. Coronaviruses, including SARS-CoV-2, use RNA-dependent RNA polymerase (RdRP) for viral replication and transcription. Since RdRP is a promising therapeutic target for infection of SARS-CoV-2, it would be beneficial to develop new experimental tools for analysis of the RdRP reaction of SARS-CoV-2. Here, we succeeded to develop novel mouse monoclonal antibodies (mAbs) that recognize SARS-CoV-2 nsp12, catalytic subunit of the RdRP. These anti-nsp12 mAbs, RdMab-2, -13, and -20, specifically recognize SARS-CoV-2 nsp12 by western blotting analysis, while they exhibit less or no cross-reactivity to SARS-CoV nsp12. In addition, SARS-CoV-2 nsp12 was successfully immunoprecipitated using RdMab-2 from lysates of cells overexpressing SARS-CoV-2 nsp12. RdMab-2 was able to detect SARS-CoV-2 nsp12 transiently expressed in established culture cells such as HEK293T cells by indirect immunofluorescence technique. These novel mAbs against SARS-CoV-2 nsp12 are useful to elucidate the RdRP reaction of SARS-CoV-2 and biological cell response against it.

**Supplementary Information:**

The online version contains supplementary material available at 10.1186/s12985-022-01948-2.

## Introduction

While human coronaviruses (CoV) are widespread in humankind as a pathogen for the common cold, severe acute respiratory syndrome CoV (SARS-CoV), Middle East respiratory syndrome CoV (MERS-CoV), and SARS-CoV-2 cause severe acute respiratory syndrome and have extremely high mortality rates [1–4]. Among them, SARS-CoV-2 emerged in Wuhan city, China at the end of 2019 and spread worldwide, causing a global pandemic of coronavirus disease 19 (COVID-19) [2, 3]. Emerging evidences have clarified the virological properties of SARS-CoV-2 and the biological mechanism by which leads to symptoms of COVID-19 [5].

SARS-CoV-2 is a positive-sense single-stranded RNA virus that has a large RNA genome (~ 30 kb) and shares high sequence homology to SARS-CoV (~ 80%) and MERS-CoV (~ 50%) [6]. A series of non-structural proteins (nsps), which is produced by cleaving two polyproteins, polyprotein1a (pp1a) and polyprotein1ab (pp1ab) translated from the genomic RNA, assemble into viral replication and transcription complexes [7]. Among these nsps, nsp12, which is the catalytic subunit of the RdRP complex containing nsp7 and nsp8 as cofactors, catalyzes the synthesis of viral RNA and thus plays a pivotal role in the viral replication and transcription [8–11], although the detailed transcription mechanism of CoVs remains unclear. Therefore, nsp12 is considered a promising therapeutic target for antiviral inhibitors such as remdesivir, which is practically utilized to treat COVID-19 [12–14]. Recent structural analysis of SARS-CoV-2 nsp12 by electron cryo-microscopy has revealed its right-hand shaped structure like RdRPs of other RNA viruses [8–11]. Intriguingly, the structures of nsp12 encoded by SARS-CoV-2 has a characteristic β-hairpin motif in the nidovirus RdRP-associated nucleotidyltransferase (NiRAN) domain, which is structurally different from that of SARS-CoV [9]. However, difference between the RdRP reactions of SARS-CoV and SARS-CoV-2 remains unclarified.

Although a few antibodies (Abs) against nsp12 of SARS-CoV-2 are commercially available, these Abs are all polyclonal and not highly specific. In addition, these polyclonal Abs cannot discriminate between nsp12 of SARS-CoV and SARS-CoV-2. To study the molecular mechanism underlying the RdRP reaction of SARS-CoV-2, monoclonal antibody (mAb) against nsp12 of SARS-CoV-2, which has not been developed so far, could be powerful tools. Furthermore, the mAb that could specifically detect SARS-CoV-2 nsp12, but not that of SARS-CoV, would be highly potential to examine difference between the RdRP reactions of SARS-CoV and SARS-CoV-2. In this study, we have established novel mouse mAbs against SARS-CoV-2 nsp12 (RdMabs), and evaluated whether they could be applied for western blotting, immunoprecipitation, and immunostaining analyses. In addition, we examined the specificity of these mAbs, especially focusing on whether they discriminate between nsp12 of SARS-CoV and SARS-CoV-2.

## Materials and methods

### Cells and antibodies

P3X63Ag8U.1 (P3U1) and SV40-transformed human embryonic kidney cell line HEK-293T (293T) cells were obtained from the American Type Culture Collection (Manassas, VA). P3U1 cells were cultured in a Roswell Park Memorial Institute (RPMI) 1640 medium (Nacalai Tesque, Inc., Kyoto, Japan) that was supplemented with 10% heat-inactivated fetal bovine serum (FBS; Thermo Fisher Scientific Inc., Waltham, MA), 100 U/mL penicillin, 100 μg/mL streptomycin, and 0.25 μg/mL amphotericin B (Nacalai Tesque, Inc.). 293T cells were cultured in Dulbecco’s modified Eagle’s medium supplemented with 10% FBS, streptomycin (100 μg/ml), and penicillin (100 U/ml).

Anti-β-actin (AC-15) mouse mAb (Merck, Darmstadt, Germany) and anti-FLAG mouse mAb (M2) (Merck) were used for western blotting analysis. mAbs against nsp12 of SARS-CoV-2 (RdMab-2, -13, and -20) were developed in this study (see below).

### Hybridoma production of mAbs against nsp12 of SARS-CoV-2

**Fig. 1:**
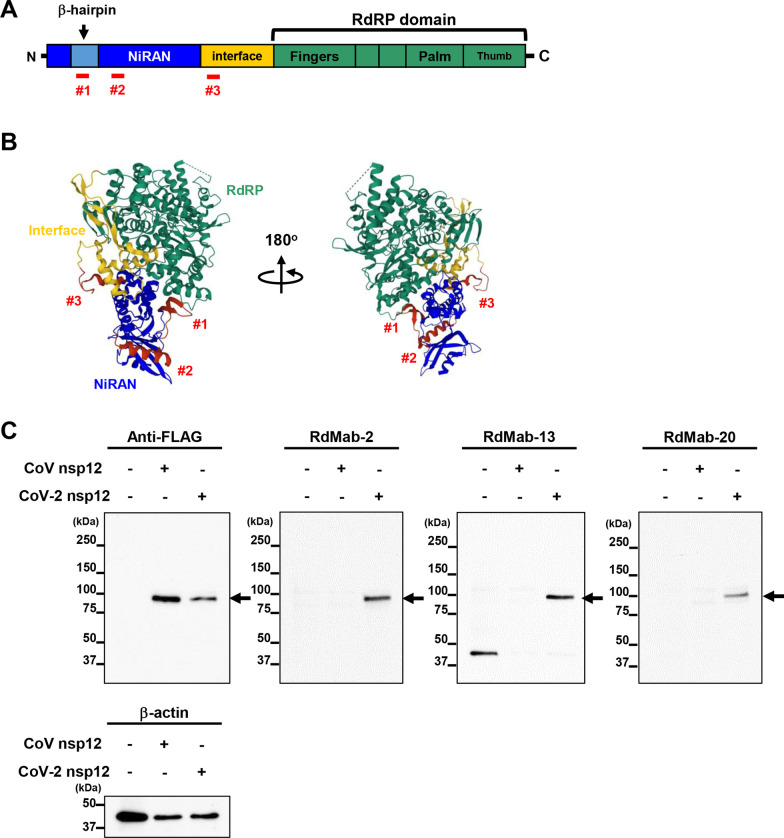
Development of mouse monoclonal antibodies against SARS-CoV-2 nsp12. **A**, **B** Structure of SARS-CoV-2 nsp12. The RdRP nsp12 comprises the NiRAN, interface, and the RdRP domains, which has the right-handed structure composed of fingers, palm, and thumb domains. The ribbon diagrams were cited from Protein Data Bank (PDB) (accession numbers 7BTF). NiRAN, the nidovirus RdRP-associated nucleotidyltransferase domain. The peptides (#1–3) used to immunize mice are shown. **C** Western blotting analysis using mouse monoclonal antibodies (mAb) against SARS-CoV-2 nsp12 (RdMab-2, -13, and -20). A FLAG-tagged nsp12 of SARS-CoV-2 transiently expressed in 293T cells was detected using an anti-FLAG antibody and RdMabs

Three female BALB/c mice (6-weeks-old) were purchased from CLEA Japan (Tokyo, Japan). The animals were housed under specific pathogen-free conditions. The Animal Care and Use Committee of Tohoku University approved all animal experiments (Permit number: 2019NiA-001). We designed three peptides (#1–3) of nsp12 as immunogens to immunize mice around the NiRAN domain (Fig. 1A, B). To develop mAbs against nsp12 of SARS-CoV-2, three synthesized peptides, such as _34_AFDIYNDKVAGFAKFLKTNC_53_, _74_RHTFSNYQHEETIYNLLKDC_83_, and _248_TRALTAESHVDTDLTKPYIC_266_, which were keyhole limpet hemocyanin (KLH)-conjugated (Eurofins Genomics, Tokyo, Japan) were immunized intraperitoneally (i.p.) with Imject Alum (Thermo Fisher Scientific Inc.) into BALB/c mice (100 μg of each peptide/one mouse). The procedure included three additional immunization procedures (100 μg of each peptide), followed by a final booster intraperitoneal injection (100 μg of each peptide) 2 days before its spleen cells were harvested. The harvested spleen cells were subsequently fused with P3U1 cells, using polyethylene glycol 1500 (PEG1500; Roche Diagnostics, Indianapolis, IN). Then, hybridomas were grown in an RPMI medium supplemented with hypoxanthine, aminopterin, and thymidine for selection (Thermo Fisher Scientific Inc.). Cultured supernatants were finally screened using enzyme-linked immunosorbent assay (ELISA) for the detection of nsp12 peptides [15]. Of 24 clones (RdMab-1 to -24), three clones (RdMab-2, 13, 20) were cultured using Hybridoma-SFM medium (Thermo Fisher Scientific Inc.), and were purified with Ab-Capcher (ProteNova, Kagawa, Japan).

### Plasmids

The plasmid vectors, pCMV-FLAG-CoV-nsp12 and -CoV-2-nsp12, containing a FLAG-tagged nsp12 expression cassette driven by a CMV promoter, was designed and constructed by VectorBuilder Inc. (Chicago, IL). The SARS-CoV and -CoV-2 nsp12 genes (GenBank: NC_004718 and MN908947, respectively) cloned into the plasmid vectors were codon optimized for expression in human cells.

### Transfection of plasmids

To overexpress SARS-CoV and -CoV-2 nsp12 transiently, 293T cells were transfected with plasmid vectors carrying a FLAG-tagged nsp12 expression cassette, pCMV-FLAG-CoV-nsp12 and -CoV-2-nsp12 using Lipofectamine 2000 (Thermo Fisher Scientific). After 72 h of incubation, cells were lysed in RIPA buffer (Nacalai Tesque) for western blotting analysis.

### Western blotting analysis

Western blotting assay was performed as previously described [16]. Briefly, whole-cell extracts were prepared and 30 μg of total protein per lane was loaded onto 4–20% gradient sodium dodecyl sulfate (SDS)-polyacrylamide gels. After electrophoresis under reducing conditions, bands of protein were transferred to nitrocellulose membranes (Amersham; Cytiva, Marlborough, MA). After blocking with 5% skim milk prepared in TBS-T (Tween-20, 0.1%), the membrane was incubated with 5 μg/mL of RdMab-2, -13, and -20, 1:5000 dilution of anti-FLAG (clone M2; Merck), or 1:10,000 dilution of anti-β-actin (clone AC-15; Merck), followed by incubation in the presence of horseradish peroxidase (HRP)-labeled anti-mouse IgG antibody (1:5000; Cell Signaling Technology, Danvers, MA).

### Immunoprecipitation assay

Immunoprecipitation assays were performed as previously described [15]. Briefly, 293T cells were transfected with a FLAG-tagged nsp12-expressing plasmid (pCMV-FLAG-CoV-2-nsp12). After 72 h of incubation, 1 × 10^7^ cells were lysed in 1 ml of Lysis buffer A (0.5% NP-40, 20 mM Tris–HCl (pH 7.4), and 150 mM NaCl). After sonication, lysates were cleared of insoluble material by centrifugation at 21,000×*g* at 4 °C for 15 min. FLAG-nsp12 proteins were co-immunoprecipitated using Pierce Protein A Plus Agarose (Thermo Fisher Scientific) and 20 µg of anti-nsp12 mAb (RdMab-2) from the lysate, and then precipitated immune complexes were eluted in 2 × SDS loading buffer (2% β-mercaptoethanol, 20% glycerol, 4% SDS, and 100 mM Tris–HCl (pH 6.8)). The eluted proteins were detected by western blotting analysis. To detect the precipitated FLAG-nsp12 proteins, anti-FLAG mouse mAb (1:5000; M2) and Mouse TrueBlot ULTRA Anti-Mouse Ig HRP (1:4000; Rockland, Gilbertsville, PA) were used for western blotting analysis.

### Immunofluorescence cell staining

Immunofluorescence cell staining was performed as previously described [16]. Briefly, cells were fixed with 4% formaldehyde in PBS, permeabilized with 0.2% TritonX-100 in PBS, and blocked with 2% bovine serum albumin in PBS. The cells were incubated with 10 μg/mL the primary antibody RdMab-2, followed by incubation in the presence of Alexa488-labeled goat anti-mouse IgG (1:1000; Thermo Fisher Scientific). The cells were mounted in ProLong Glass Antifade Mountant with NucBlue Stain (Thermo Fisher Scientific) and imaged under a fluorescent microscope (IX81, Olympus, Tokyo, Japan).

## Results

### Establishment of anti-nsp12 mAbs

Structural analyses using electron cryo-microscopy have shown that SARS-CoV-2 nsp12 protein has a closed, right-handed structure, which consists of palm, finger, and thumb domains (Fig. 1A, B) [8, 9]. Comparative analysis of structures of nsp12 encoded by SARS-CoV and SARS-CoV-2 suggested that SARS-CoV-2 nsp12 has a characteristic β-hairpin motif in the NiRAN domain, which is structurally different from that of SARS-CoV [9]. We hypothesized that antibodies recognizing the NiRAN domain in SARS-CoV-2 nsp12 could discriminate between nsp12 of SARS-CoV and SARS-CoV-2. Therefore, we designed and utilized three peptides (#1–3) of nsp12 around the NiRAN domain, but not recombinant nsp12 protein, as immunogens to immunize mice (Fig. 1A, B) and subsequently established hybridomas producing anti-nsp12 mAbs from the mouse spleen. We first screened the culture supernatants by ELISA for binding to nsp12 peptides, which selected 24 mAb clones (RdMab-1-24) (Table 1). To examine whether these putative clones binds to the nsp12 protein, we transiently expressed FLAG-tagged nsp12 of SARS-CoV-2 in 293T cells and then performed western blotting analysis using the culture supernatants of hybridomas producing these clones. We confirmed that anti-FLAG mAb (M2) recognized the FLAG-tagged nsp12 of SARS-CoV-2 in the position of the predicted size (~ 100 kDa) (Additional File 1: Fig. S1). The screening of 24 mAb clones by western blotting analysis demonstrated that 6 clones (RdMab-1, -2, -13, -15, -16, and -20) could detect SARS-CoV-2 nsp12 (Additional File 1: Fig. S1). To further evaluate whether they discriminate between nsp12 of SARS-CoV and SARS-CoV-2, we performed western blotting analysis using 293T cells transiently expressing the FLAG-tagged nsp12 of SARS-CoV or SARS-CoV-2, suggesting that the culture supernatants of three clones (RdMab-2, -13, and -20) could specifically detect SARS-CoV-2 nsp12 (Additional File 1: Fig. S2). Next, we purified these clones (RdMab-2, -13, and -20) from the hybridoma supernatants, which recognized SARS-CoV-2 nsp12 but not that of SARS-CoV (Fig. 1C). Together, we established three clones (RdMab-2, -13, and -20) which could specifically detect SARS-CoV-2 nsp12.

**Table 1:** Subclass of RdMabs

Clone	Immunogen	Animal	Subclass	
			HC	LC
RdMab-1	#2, RHTFSNYQHEETIYNLLKDC	Mouse	IgG_1_	Kappa
RdMab-2	#3, TRALTAESHVDTDLTKPYIC	Mouse	IgG_2a_	Kappa
RdMab-3	#1, AFDIYNDKVAGFAKFLKTNC	Mouse	IgM	Kappa
RdMab-4	#2, RHTFSNYQHEETIYNLLKDC	Mouse	IgG_1_	Kappa
RdMab-5	#2, RHTFSNYQHEETIYNLLKDC	Mouse	IgG_1_	Kappa
RdMab-6	#3, TRALTAESHVDTDLTKPYIC	Mouse	IgG_1_	Kappa
RdMab-7	#1, AFDIYNDKVAGFAKFLKTNC	Mouse	IgG_1_	Lambda
RdMab-8	#2, RHTFSNYQHEETIYNLLKDC	Mouse	IgG_1_	Kappa
RdMab-9	#2, RHTFSNYQHEETIYNLLKDC	Mouse	IgG_1_	Kappa
RdMab-10	#2, RHTFSNYQHEETIYNLLKDC	Mouse	IgG_1_	Kappa
RdMab-11	#3, TRALTAESHVDTDLTKPYIC	Mouse	IgM	Kappa
RdMab-12	#3, TRALTAESHVDTDLTKPYIC	Mouse	IgG_1_	Kappa
RdMab-13	#3, TRALTAESHVDTDLTKPYIC	Mouse	IgM	Kappa
RdMab-14	#2, RHTFSNYQHEETIYNLLKDC	Mouse	IgG_1_	Kappa
RdMab-15	#2, RHTFSNYQHEETIYNLLKDC	Mouse	IgG_1_	Kappa
RdMab-16	#2, RHTFSNYQHEETIYNLLKDC	Mouse	IgG_1_	Kappa
RdMab-17	#3, TRALTAESHVDTDLTKPYIC	Mouse	IgG_1_	Kappa
RdMab-18	#1, AFDIYNDKVAGFAKFLKTNC	Mouse	IgG_1_	Lambda
RdMab-19	#1, AFDIYNDKVAGFAKFLKTNC	Mouse	IgG_1_	Lambda
RdMab-20	#3, TRALTAESHVDTDLTKPYIC	Mouse	IgG_2b_	Kappa
RdMab-21	#3, TRALTAESHVDTDLTKPYIC	Mouse	IgM	Kappa
RdMab-22	#3, TRALTAESHVDTDLTKPYIC	Mouse	IgG_1_	Kappa
RdMab-23	#1, AFDIYNDKVAGFAKFLKTNC	Mouse	IgM	Kappa
RdMab-24	#1, AFDIYNDKVAGFAKFLKTNC	Mouse	IgG_1_	Lambda

### Application to immunoprecipitation assay and immunofluorescence cell staining

**Fig. 2:**
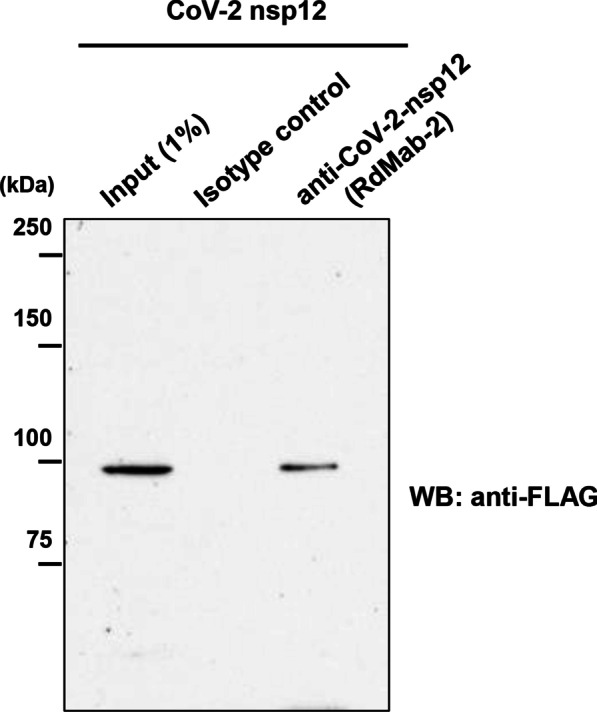
Immunoprecipitation of SARS-CoV-2 nsp12 using RdMab-2. SARS-CoV-2 nsp12 proteins were immunoprecipitated by RdMab-2 from 293T cells transiently expressing a FLAG-tagged nsp12 of SARS-CoV-2, and then detected by an anti-FLAG antibody. Mouse IgG_2a_ was used as an isotype control for the immunoprecipitation

**Fig. 3:**
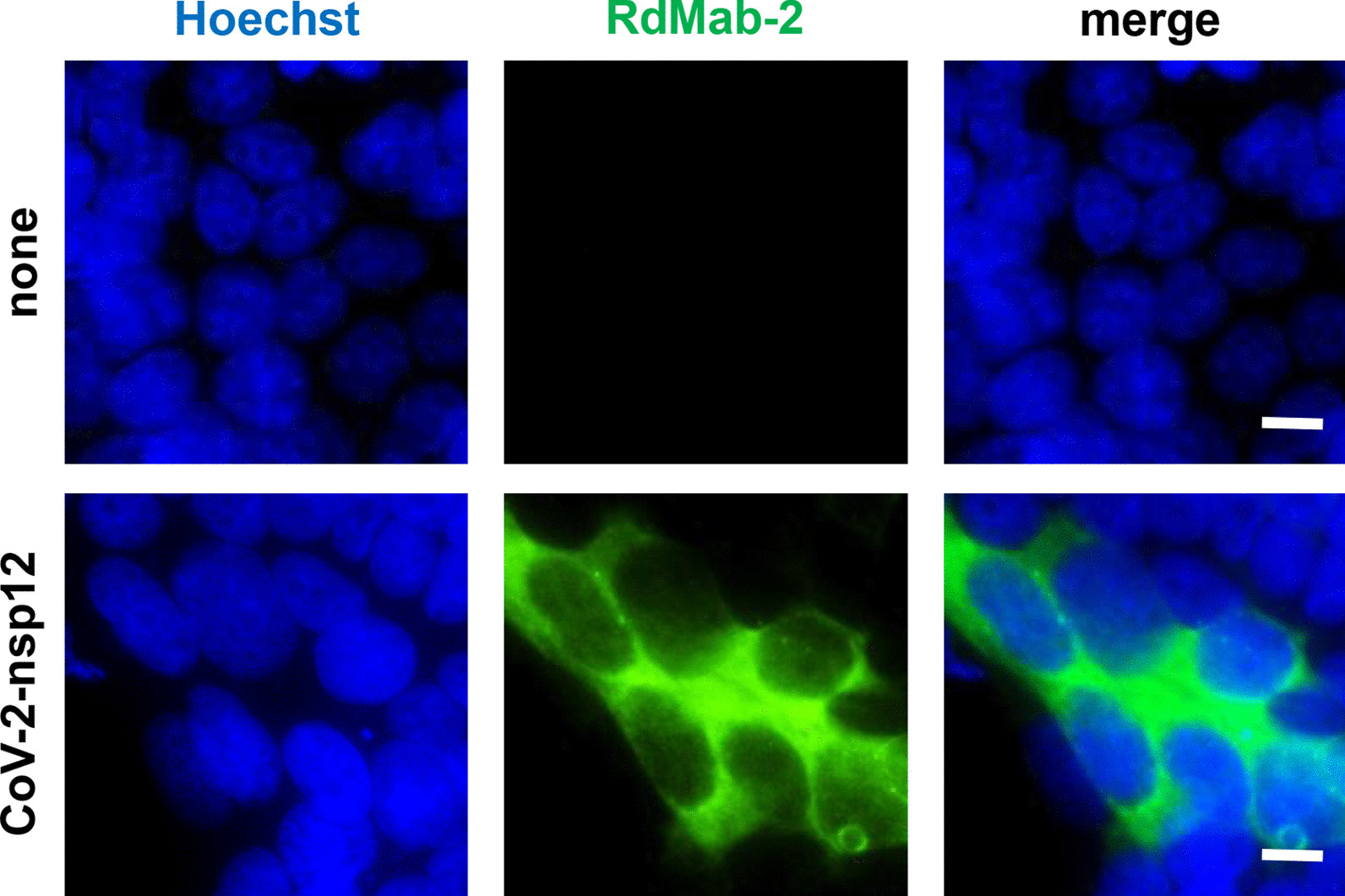
Immunofluorescence imaging of SARS-CoV-2 nsp12 using RdMab-2. 293T cells transiently expressing a FLAG-tagged nsp12 protein of SARS-CoV-2 were immunostained with RdMab-2. Scale bar: 10 μm

We next performed immunoprecipitation assay using lysates of 293T cells transiently expressing the FLAG-tagged nsp12 of SARS-CoV-2. Immunoprecipitation with RdMab-2 successfully isolated the FLAG-tagged nsp12 of SARS-CoV-2 from the lysates (Fig. 2), whereas RdMab-13 and -20 failed to isolate it by immunoprecipitation (data not shown). We confirmed using an isotype matched irrelevant control that the immune-complex precipitated by the RdMab-2 is specific signal (Fig. 2). Finally, we applied RdMab-2 to immunostaining analysis. No signals were detected in non-transfected 293T cells (Fig. 3, upper). When we transiently overexpressed the nsp12 in 293T cells, we found discrete signal of nsp12, which was spreading throughout the cytoplasm (Fig. 3, lower). This results are consistent with previous reports that the RdRP reaction of CoVs, including SARS-CoV, is conducted in the cytoplasm [17–19]. Together, these findings indicated the utility of RdMab-2 for immunoprecipitation and immunofluorescence cell staining assays.

## Discussion

The recent global pandemic of SARS-CoV-2 has caused catastrophic consequences [2, 3]. The research about infection with SARS-CoV-2 and the development of therapeutic agents for COVID-19 is ongoing. Among several SARS-CoV-2 proteins, its RdRP protein, nsp12, is one of the crucial therapeutic targets, because it plays essential roles in viral RNA replication and transcription [7, 14]. In this study, we have established three novel mouse mAbs against SARS-CoV-2 nsp12 (RdMab-2, -13, and -20). They all detected SARS-CoV-2 nsp12 by western blotting analysis more specifically than that of SARS-CoV (Fig. 1). In addition, RdMab-2 were also suitable for immunoprecipitation (Fig. 2) and immunostaining analyses (Fig. 3).

Both SARS-CoV and SARS-CoV-2 use RdRP for viral replication and transcription, although the detailed transcription mechanism of CoVs remains unclear. It is assumed that the RdRP complex of SARS-CoV-2 executes two types of transcription; continuous transcription of the genomic RNA (gRNA) and discontinuous transcription of various sizes of subgenomic RNAs (sgRNAs) [20, 21]. Regarding the discontinuous transcription, it is hypothesized that the RdRP can skip to the 3'-end leader sequence of the template gRNA from transcription-regulatory sequences (TRSs) located in the middle of gRNA [6, 20, 22, 23], although there has been no experimental evidence for the transcription mechanism of CoVs, including SARS-CoV-2. In addition, difference between the RdRP reactions of SARS-CoV and SARS-CoV-2 remains unclarified. Therefore, the mAb that could distinguish nsp12 from SARS-CoV and SARS-CoV-2 would be potentially valuable to investigate difference between the RdRP reactions of SARS-CoV and SARS-CoV-2. RdMab-2 developed in the present study may contribute to the elucidation of this mechanism, because it has the potential for various biochemical and cell biological experiments.

While several rabbit polyclonal antibodies against nsp12 of SARS-CoV-2 are now commercially available from Cell Signaling Technology, Proteintech, GeneTex, and so on, no mAb against nsp12 of SARS-CoV-2 have been reported. Notably, Yamada et al. [24] reported that the mAb against nsp12 of SARS-CoV (clone 4E6; Novus Biologicals, Centennial, CO) can recognize nsp12 of SARS-CoV-2, although the signals from western blotting analysis are faint. Since nsp12 of SARS-CoV and SARS-CoV-2 shares high amino acid sequence homology (~ 96%) (GenBank: NC_004718 and MN908947), it seems that the mAb against nsp12 of SARS-CoV (clone 4E6; Novus Biologicals, Centennial) can recognize nsp12 of both SARS-CoV and SARS-CoV-2. We now found that RdMab-2, -13, and -20 could specifically detect the nsp12 of SARS-CoV-2 by western blotting analysis (Figs. 1, 2 and 3). These novel anti-nsp12 mAbs would be the first mAb specialized in detection of SARS-CoV-2 nsp12.

In this study, we designed three peptides (#1–3) for producing mAbs, focusing on the structure around the NiRAN domain in the nsp12, because SARS-CoV-2 nsp12 has a characteristic β-hairpin motif in the NiRAN domain, which was not found by structure analysis of SARS-CoV nsp12 [9]. Intriguingly, the established mAbs (RdMab-2, -13, and -20) were all obtained from mice immunized with the same peptide #3 (Table 1). The amino acid sequence of the peptide #3 derived of SARS-CoV-2 nsp12 had lower homology (5 mutations/19 amino acids) to the corresponding sequence of SARS-CoV nsp12 than that of peptides #1 and #2 (1 mutation and 2 mutations/19 amino acids). In addition, RdMab-2, -13, and -20 preferentially recognized nsp12 of SARS-CoV-2 rather than that of SARS-CoV (Fig. 1C). These results suggests that the structure surrounding peptide #3 in the NiRAN domain might be unique to SARS-CoV-2 nsp12.

Taken together, we have successfully developed three novel mouse mAbs against SARS-CoV-2 nsp12 (RdMab-2, -13, and -20), which are suitable for western blotting, immunoprecipitation, and immunostaining analyses. These novel anti-nsp12 mAbs were able to discriminate between nsp12 of SARS-CoV and SARS-CoV-2. Generally, elucidation of specific protein functions critically depends on the ability to recognize the target protein specifically, and we obtained mAbs that could specifically recognize SARS-CoV-2 nsp12. These novel anti-nsp12 mAbs could be utilized for investigation of the RdRP reaction of SARS-CoV-2 and responses against it in host cells. In this study, we could develop mAbs against SARS-CoV-2 nsp12 as an initial step. We hope that further studies using these anti-nsp12 mAbs will lead to elucidation of the RdRP reactions of SARS-CoV-2 and development of more effective anti-viral strategies.

## Supplementary Information

**Additional file1**. **Figure S1**: Screening of anti-nsp12 mouse monoclonal antibodies using the culture supernatant of hybridomas. **Figure S2**: Selection of anti-nsp12 mouse monoclonal antibodies to discriminate between nsp12 of SARS-CoV and SARS-CoV-2.

## Data Availability

All data generated or analyzed during this study are included in this published article.
