# Supplementary material for: Development of novel monoclonal antibodies against nsp12 of SARS-CoV-2
Source: Virol J. 2022 Dec 10;19:213. doi: 10.1186/s12985-022-01948-2 (PMC9736705; doi:10.1186/s12985-022-01948-2)
Supplement: Supplementary file 1 — Additional file1. Figure S1: Screening of anti-nsp12 mouse monoclonal antibodies using the culture supernatant of hybridomas. Figure S2: Selection of anti-nsp12 mouse monoclonal antibodies to discriminate between nsp12 of SARS-CoV and SARS-CoV-2. [file 12985_2022_1948_MOESM1_ESM.pdf]

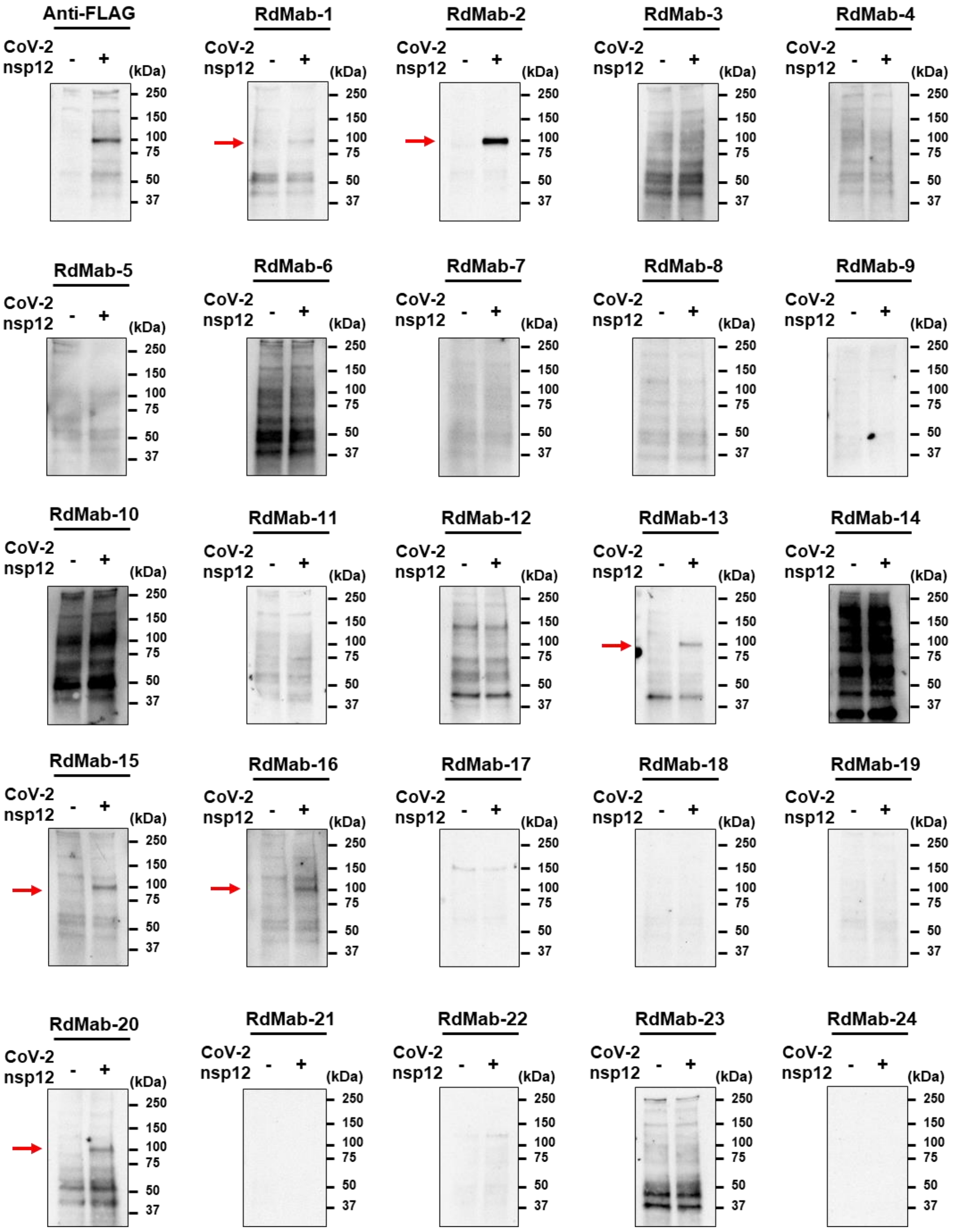

Fig. S1. Screening of anti-nsp12 mouse monoclonal antibodies using the culture supernatant of hybridomas

Western blotting analysis to detect a FLAG-tagged nsp12 of SARS-CoV-2 transiently expressed in 293T cells. The FLAG-tagged nsp12 was detected using an anti-FLAG antibody (M2) and the culture supernatant of hybridomas.

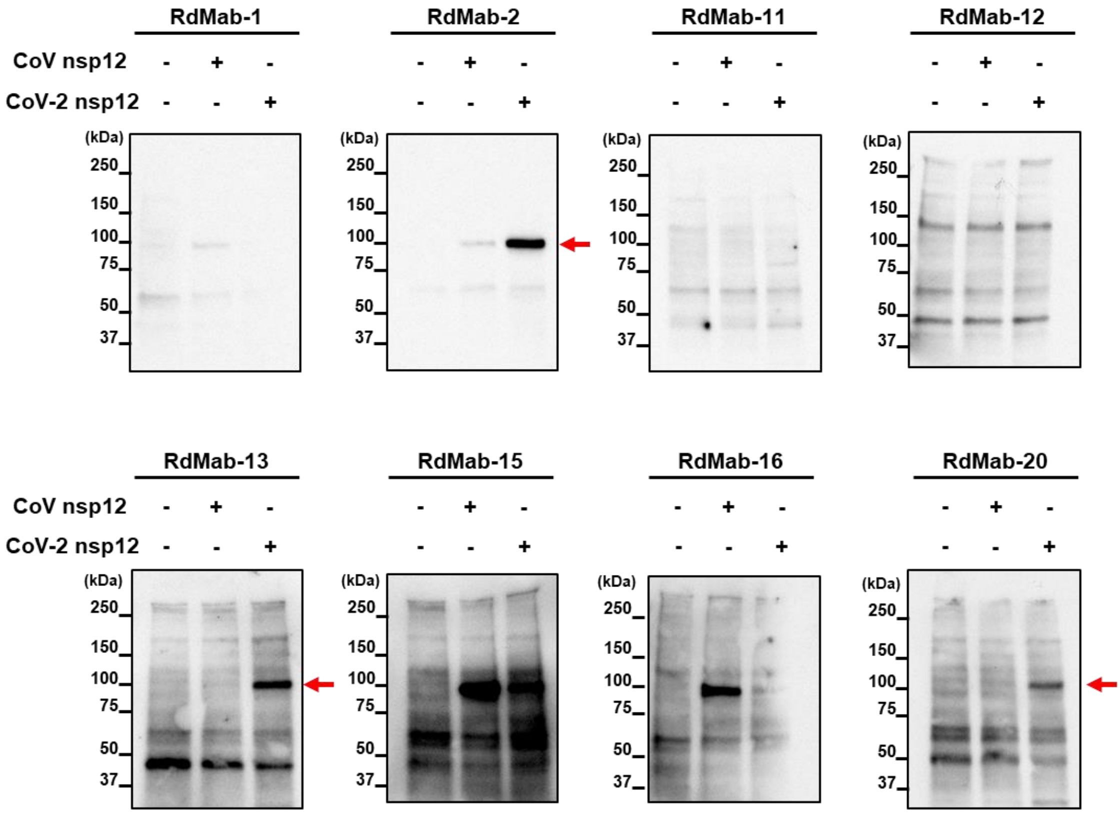

Fig. S2. Selection of anti-nsp12 mouse monoclonal antibodies to discriminate between nsp12 of SARS-CoV and SARS-CoV-2

Western blotting analysis to detect a FLAG-tagged nsp12 of SARS-CoV and SARS-CoV-2 transiently expressed in 293T cells. The FLAG-tagged nsp12 was detected using the culture supernatant of hybridomas.
